# Using Technology to Assess Bidirectionality between Daily Pain and Physical Activity: The Role of Marginalization during Emerging Adulthood

**DOI:** 10.3390/children8090756

**Published:** 2021-08-30

**Authors:** Helen Bedree, Steven A. Miller, Joanna Buscemi, Rachel Neff Greenley, Susan T. Tran

**Affiliations:** 1 Department of Psychology, DePaul University, Chicago, IL 60614, USA; jbuscem2@depaul.edu (J.B.); susan.tran@depaul.edu (S.T.T.); 2 Department of Psychology, Rosalind Franklin University of Medicine and Science, North Chicago, IL 60064, USA; steven.miller@rosalindfranklin.edu (S.A.M.); rachel.greenley@rosalindfranklin.edu (R.N.G.)

**Keywords:** emerging adults, pain symptoms, physical activity, marginalized status, longitudinal, ActiGraph, dynamic structural equation modeling

## Abstract

Emerging adulthood is often overlooked as a developmental time period critical to shaping future health outcomes. Recurrent pain is a commonly experienced health concern within this age group, particularly headaches and low back pain, and early experiences of recurrent pain are related to subsequent chronic pain and disability. Furthermore, adults from marginalized populations report more frequent and severe recurrent pain. Many studies have demonstrated the therapeutic effect of physical activity on pain relief; however, others have demonstrated that physical activity can also exacerbate pain symptoms. Therefore, the current study aimed to (1) assess a bidirectional relationship between reported pain and engagement in physical activity among an emerging adult sample (*N* = 265) and (2) determine whether sociodemographic factors moderate this relationship. Using longitudinal daily reported pain and ActiGraph monitor data collected over two weeks, a novel dynamic structural equation modeling approach was employed. Results indicated no significant cross-lagged relationships between pain and physical activity, and no significant moderation effects. These findings suggest that a bidirectional relationship does not exist among a diverse college sample of emerging adults even after considering sociodemographic moderators. Excellent retention and few missing data suggest that using accelerometers and daily diaries are feasible methods to collect data in this population. Sample considerations and future analytical approaches are discussed.

## 1. Introduction

Emerging adulthood (ages of 18–25 years) is an important developmental context for shaping health outcomes [1], with new opportunities for autonomy and decision making in health behaviors [2]. Adopting health behaviors has significant implications for the development of chronic conditions during this developmental phase [3]. Recurrent pain is a health concern of particular importance given most emerging adults (65%) report pain-related symptoms [4]. Low back pain is prevalent among this age group [5,6] with 71% reporting low back pain one to five times a week [7]. Headaches are another common pain symptom reported by emerging adults, with the majority (57% male, 78% female) experiencing their most recent headache in the past month [8]. Many students (78%) indicated that their headaches affect daily life, such as leading to worse overall academic performance [9]. With the prevalence of pain conditions rising [10], it is critical to understand health behaviors that can prevent a chronic pain trajectory, which is associated with numerous psychosocial and functional limitations and, overall, lower health-related quality of life [11].

Physical activity is one health-promoting behavior that has been found to reduce pain symptomatology [12,13]. Research has found a therapeutic link between physical activity and pain relief among children and adults with chronic pain in clinical and non-clinical settings [14,15,16]; for example, thirty minutes of moderate exercise one to three times a week was associated with a 10–12% lower prevalence of chronic pain among adults in a population-based study [17]. According to a research study conducted by the American College Health Association (2013), however, most emerging adults are not engaging in enough physical activity. Only 18% of this population are engaging in moderate-intensity physical activity at least 5 days per week and 26% are meeting the standards for vigorous-intensity at least 3 days per week, with a total of 45% meeting either the moderate-intensity or vigorous-intensity 2007 physical activity guidelines [18]. Yahia and colleagues (2015) discovered similar results, with only approximately half of their college sample engaging in some form of reported physical activity and one-quarter endorsing an “active lifestyle” [19]. Racette and colleagues (2008) reported that one-quarter of their college student sample did not engage in any exercise [20], similar to the U.S. adult population [21].

In the relationship between pain and physical activity, it is important to consider that there may be a threshold or bidirectional relationship. For example, while research has found that physical activity can reduce pain [14,15,16], individuals with chronic pain have also reported having a decreased ability to engage in physical activity [22,23], suggesting a bidirectional relationship between pain and physical activity. Essentially, while physical activity can ameliorate pain symptoms, pain can also limit physical functioning. Rabbitts and colleagues (2014) found that higher pain intensity predicted next-day lower peak physical activity for adolescents with chronic pain and healthy matched controls, while higher physical activity predicted end of day lower pain only for youth with chronic pain. Given the mixed findings, it is important to better understand this complex relationship among emerging adults with and without frequent pain symptoms to provide a framework for preventing and managing pain among this developmental group.

To fully understand the relationship between pain and physical activity among emerging adults, it is critical to consider how identity factors, such as race and ethnicity, gender, and socioeconomic status (SES) influence health outcomes and behaviors. A growing body of research demonstrates the deleterious relationship between marginalized social status and well-being [24], taking form in health inequities, which are preventable and unjust [25]. Upstream factors, or those at the root of health inequities, such as economic and social resources, educational attainment, living and working conditions, and systematic oppression have been shown to be causally linked to downstream factors, or those most often attributed in explaining health outcomes, like behavior and health care utilization [24]. Research has shown that pain is inequitably experienced by different sociodemographic groups, with worse pain experiences (frequency and severity; greater risk for pain related disability) among Latinx and Black/African American adults with chronic pain, women, and those with lower SES [8,26,27,28,29,30,31,32,33,34]. Further, Black/African American and Hispanic adults, women, and those with low SES report lower rates of physical activity [19,35,36,37,38] than their white, male, and higher SES counterparts.

Taken together, these studies demonstrate how social determinants of health inequitably impact pain and physical activity. Upstream factors, such as chronic minority stress due to prejudice and discrimination, are linked to worse health outcomes [39] and likely exacerbate experiences of pain among individuals who are marginalized. Individuals with low SES may have fewer resources (limited access to nutrition, and safe and affordable physical activity resources [24]) to facilitate coping with this health concern compared to non-marginalized individuals who do not face additional stress and have resources to cope with any health concerns. Therefore, it is possible that marginalization may moderate and exacerbate the relationships between pain and physical activity. No study to our knowledge has tested the moderation effects of race, ethnicity, gender, and SES on the relationship between pain and physical activity.

Traditional self-report, retrospective pain and physical activity assessments have methodological weaknesses, such as recall bias. Objective technological measures and daily diary data collection can better assess these constructs. Implementing technological advances is particularly helpful when working with emerging adults, as this population has been uniquely identified for their technology use in many aspects of their lives, such as in social, personal [40], and academic contexts [41]. Use of technological tools is critical when considering accuracy and feasibility of data collection. For example, daily diaries are able to collect moment-by-moment data on symptomatology compared to retrospective self-reports collected after a study period. Further, objective measures of physical activity, such as Actigraphy monitors, also allow for increased accuracy and feasibility of data collection. They have been adapted to make data collection more feasible; research has shown that adolescents are more likely to wear wrist-worn accelerometers versus waist-worn accelerometers due to increased comfortability and decreased social factors (e.g., feeling embarrassed with waist placement) [42].

This study aimed to determine whether experiences of pain recorded by daily diary and accelerometer-measured physical activity are bidirectionally related. It was hypothesized that previous-day moderate to vigorous physical activity (MVPA) is expected to predict next-day pain, such that higher rates of MVPA will result in lower reported pain, in line with the majority of research demonstrating the therapeutic effect of physical activity in managing pain. Additionally, given past findings that there may be a bidirectional relationship between pain and physical activity among those with and without chronic pain [43], the current study also expected that previous-day pain will predict next-day MVPA, such that higher rates of pain will result in lower observed MVPA. The second aim of the current study examined whether marginalization (race, ethnicity, gender, and socioeconomic status) moderates these bidirectional relationships, due to findings demonstrating the inequitable disparities in experiencing worse pain and lower levels of physical activity, such that marginalization will exacerbate these relationships and demonstrate a stronger link between physical activity and pain.

## 2. Materials and Methods

### 2.1. Participants

Emerging adults (*N* = 265) who took part in a larger study aiming to understand health behaviors were included in the current study. The larger study has examined bidirectional health symptoms and daily hassles among emerging adults, in addition to the relationships between disease factors and social support among university students with chronic illness. Participants were enrolled at a private university in a Midwestern, diverse city in the United States. Many students are first-generation college students and receive financial aid to facilitate their tuition, and therefore this sample is more diverse in these cultural and demographic characteristics than other university samples. Participants were on average 19.61 years old (*SD* = 1.42), approximately half identified as white (53.20%), approximately two-thirds female (69.10%), majority non-Hispanic (76.60%), and reporting a parent income above $50,000 (73.96%). See Table 1 for full sample characteristics.

Eligibility requirements for the larger study included the following: age of 18–24 years old at the time of recruitment, current enrollment at the university as an undergraduate student, fluency in English to complete study questionnaires, access to a mobile phone with unlimited text-messaging for daily assessments, and non-varsity athlete status due to differential likelihood of time spent exercising. Participants were recruited from posted flyers around campus, in-person recruitment from student groups and approved classes, and from other participants who were provided a flyer with study contact information.

### 2.2. Procedure

Participants completed a baseline assessment in-person on a tablet or desktop computer, measuring behaviors relating to health promotion and risk. Participants were then followed daily for two weeks. Researchers sent text messages at agreed upon times in the morning to remind participants to wear the activity monitor, as well as at night to complete daily diary measures of hassles and physical symptoms. Participants completed daily diary measures electronically through Qualtrics software. Responses were accepted if received between 7 p.m. and 2 a.m. The research team deemed any survey sent outside of these parameters not to be an accurate representation of daily experiences within the past 24 h. Participants were asked to wear a wrist ActiGraph monitor (ActiGraph wGT3X-BT, Pensacola, FL) during the two-week period to measure daily activity. At the end of the two-week period, participants returned to the lab for an in-person follow-up assessment and returned the ActiGraph. In accordance with IRB requirements, all participants received information on the study procedure and provide informed consent prior to participating. The current study was approved by the Institutional Review Boards of DePaul University (IRB#ST042717PSY) and Rosalind Franklin University of Medicine and Science (IRB#CHP17-017). Following the completion of all study tasks, participants were compensated for completing baseline and follow-up measures ($15 gift card and $20 gift card, respectively) and for each daily survey ($5 gift card each, sum paid weekly). 

### 2.3. Measures

#### 2.3.1. Pain

Pain was measured as a composite score of self-report pain items from the Physical Health Questionnaire-15 (PHQ-15). The PHQ-15 is a brief, 15-item questionnaire assessing a variety of physical health symptoms. Past research has found high internal consistency (α = 0.80) for the complete PHQ-15 scale among primary care and obstetrics-gynecology samples [44]. The current study includes different pain symptoms in its proposed analysis that are commonly experienced by an undergraduate population. Five items from the PHQ-15 were used for this study and include whether a participant has experienced stomach pain, back pain, pain in arms, legs, or joints, headaches, and chest pain and how much they were bothered by this symptom on a three-point Likert-type scale (0 = “Not bothered at all”, 1 = “Bothered a little”, or 2 = “Bothered a lot”). Average daily composite scores were used from the two-week study period and the possible range of scores is 0 to 10 for the five items included in this study. Previous studies have used the same pain cluster of items to assess pain location [45]. Measures of internal consistency for the five-item pain subscale at baseline in the current study are poor (α = 0.55). The low internal consistency for the current non-chronic pain community sample is not surprising, as it would be unlikely that most participants are experiencing pain at multiple locations. Among a sample of undergraduate emerging adults, Lester and colleagues (1994) found participants reported an average of pain symptoms at 2.4 locations out of 9 possible. The dynamic structural equation model intra-class correlation (ICC) [46] for pain was 0.53, which indicates a need to account for nesting within clusters. ICC provides information about the amount of variance in the momentary assessment of a construct associated with the nesting unit (within-individual).

#### 2.3.2. Physical Activity

Participants were asked to wear an ActiGraph monitor continuously for the full two-week study period and until their follow-up appointment (approximately 14 days). Participants were instructed to wear the monitor on their non-dominant hand and only remove it when showering or swimming. The ActiGraph objectively measures bodily movement—activity and inactivity and energy expenditure. ActiGraph monitors measure any movement made during the time the device is worn. Raw data from each monitor was computed into length of time (minutes) in moderate to vigorous physical activity (MVPA; a composite of moderate, vigorous, and very vigorous physical activity) per day using ActiLife (v6.13.4) software. Data was transformed according to metabolic rate criteria set by Freedson and colleagues (1998) [47], in which activity counts per minute were defined for sedentary (0 to 99 activity counts), light (100 to 1951), moderate (1952 to 5724), vigorous (5725 to 9498), and very vigorous (>9499) were used. Total minutes spent in MVPA was selected for analysis in order to compare our study sample with other emerging adult samples on meeting national guideline criteria of physical activity engagement. The dynamic structural equation model ICC [46] for physical activity was 0.65, which, similar to pain, indicates a need to account for nesting within clusters. Non-wear time was detected by the ActiGraph sensor. It was excluded from computed physical activity data, as a selected feature of ActiLife (v6.13.4) software.

#### 2.3.3. Marginalized Status

Marginalized status was measured by demographic items included in the baseline assessment, specifically, race, ethnicity (Hispanic or non-Hispanic), gender, and SES (parent income) variables. A dummy coding scheme was implemented for analyses in which non-marginalized groups (white, non-Hispanic, male, and mid to high SES ($50,000 and up)) were compared with marginalized groups (person of color, Hispanic, female, and low SES ($49,999 and below)). The current delineation of low SES ($49,999 and below) was selected based on thresholds calculated by the department of Housing and Urban Development (HUD), in which very low income and low income (50% and 80% of the area median family income) in Chicago are $44,550 and $71,300, respectively, for a household of four [48]. Parent income was collected in categorical ranges (e.g., $25,000–$49,999) at baseline and the cutoff for low SES was selected consistent with the economic hardship for a family of four in this metropolitan area. Regarding gender, individuals who identified with the following options “other”, “gender non-conforming”, or “queer” (*n* = 6) were not included in the dummy-coded gender analyses due to the small sample size and possible misrepresentation; however, it is recognized that these individuals experience marginalization [49] and should be represented in future studies.

### 2.4. Statistical Analyses

**Figure 1:**
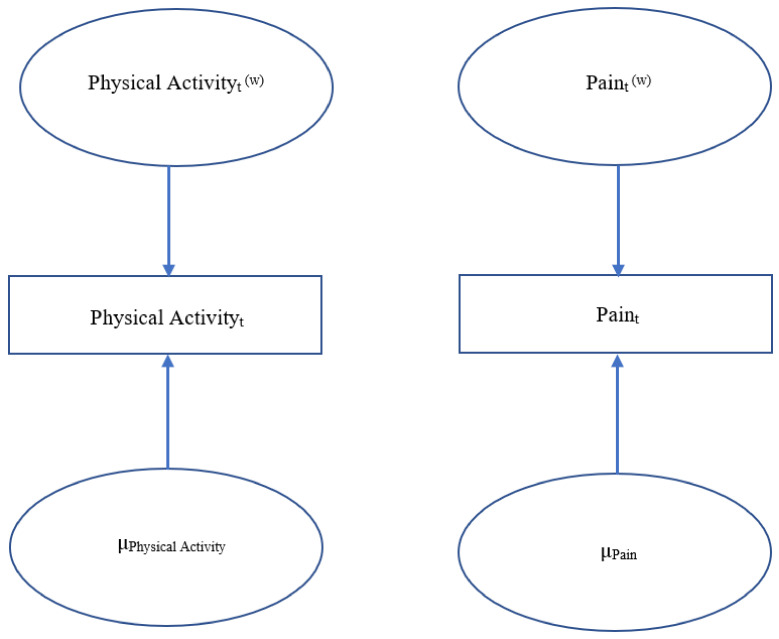
VAR(1) Model: Decomposition. *Note.* Figure 1 depicts the decomposition of the study data into within- and between-person components. T represents time point, w represents within-person, and µ represents between-person means.

**Figure 2:**
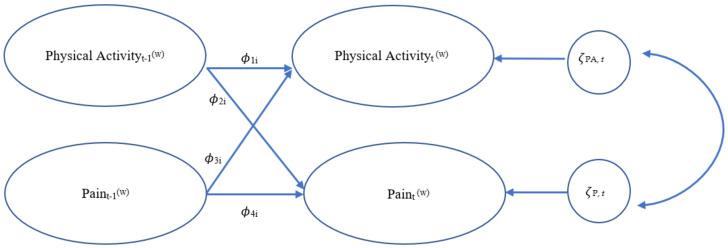
VAR(1) Model: Within-Person. *Note.* Figure 2 depicts the within-person components of the study model. T represents time point, t−1 represents previous time point, ϕ represents autoregressive parameters (how quickly an individual restores to equilibrium) and cross-lagged regression effect (predictive relationships/spill-over), PA represents physical activity, P represents pain, and ζ represents dynamic errors.

**Figure 3:**
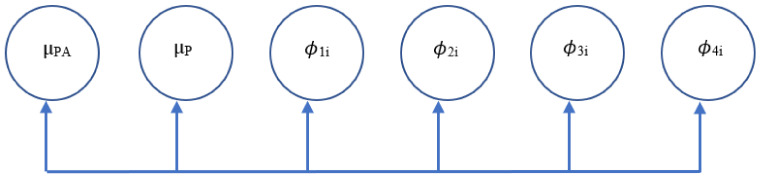
VAR(1) Model: Between-Person. *Note*. Figure 3 depicts the between-person components of the study model. PA represents physical activity, P represents pain, µ represents between-person means, and ϕ represents autoregressive parameters.

Dynamic structural equation modeling (DSEM) was used to examine the relationships between total minutes spent in MVPA per day, daily pain composite scores, and marginalized status. DSEM is a type of time series analysis that allows for comparison of intensive longitudinal data within and between individuals at multiple time points [50]. A first-order vector autoregressive VAR(1) model was used to analyze the concepts of interest. This type of model allows a vector at one time point *t* to be regressed onto the vector at a previous time point, permitting the analysis of time effects within individuals [51]. The VAR(1) model can also compare mean differences between multiple individuals. The current study examined the cross-lagged regression coefficients between daily MVPA and daily pain composite scores within individuals at time point *t* and *t*−1 to assess for strength in predictive ability. Cross-lagged parameters allow for possible determination of causal mechanisms between vectors, representing the effects of one domain of functioning onto the other [51]. DSEM has been used with this sample previously assessing for the relationships among daily hassles and physical health and an in-depth explanation of this innovative statistical approach has been provided there [52]. The current study provides a novel way of looking at the relationship between objective ActiGraph and survey data. An evaluation of these cross-lagged relationships would allow for potential causality at a given time of the effects of previous-day pain on next-day MVPA and previous-day MVPA on next-day pain, ultimately providing evidence for a bidirectional relationship. Figure 1, Figure 2 and Figure 3 depict the three features of the VAR(1) model, showing the decomposition of the study data into within- and between-person components (Figure 1), within-person model (Figure 2), and between-person model (Figure 3).

**Figure 4:**
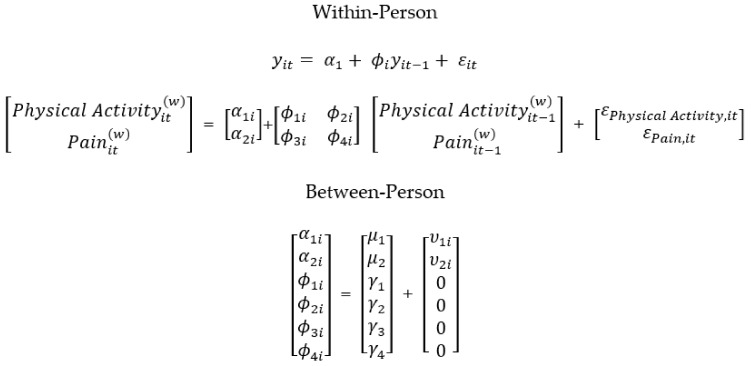
VAR(1) Model: Within-Person and Between-Person Equations.

**Figure 5:**
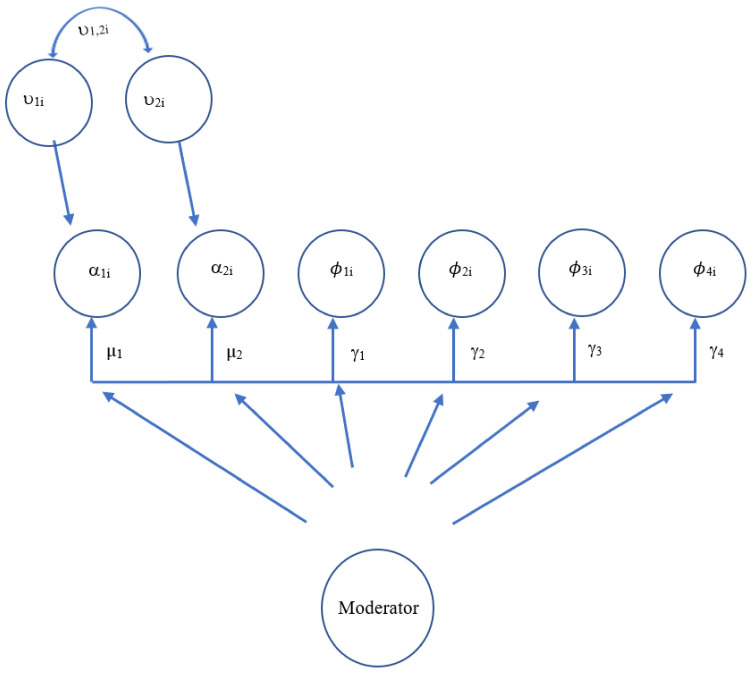
VAR(1) Model: Moderation of the Between-Person Model (Within-Person Means, Autoregressive Parameters, and Cross-Lagged Parameters). *Note.* Figure 5 depicts the moderation of the between components of the study model. **α** represents person-specific intercepts for physical activity (**α_1i_**) and pain (**α_2i_**); fixed effects (**µ_1_**, **µ_2_**); **υ_1,2i_** represents random effects related to physical activity and pain; ϕ_**1-4i**_ represents autoregressive parameters and cross-lagged regression effects; **γ_1-4_** represents fixed effects of autoregressive and cross-lagged regression parameters.

As seen in Figure 2, the within-person model demonstrates the autoregressive relationships of the vectors of interest: MVPA (PA_t_ ^(w)^) and pain (P_t_ ^(w)^) being autoregressed onto themselves on the previous day (PA_t−1_ ^(w)^) and (P_t−1_ ^(w)^), respectively (paths ϕ_1i_ and ϕ_4i_); in addition to the cross-lagged relationships of MVPA (PA_t_ ^(w)^) on previous-day pain (P_t−1_ ^(w)^) (path ϕ_3i_) and pain (P_t_ ^(w)^) on previous-day MVPA (PA_t−1_ ^(w)^) (path ϕ_2i_). These auto-regressive and cross-lagged parameters in the within-subjects model then become possible predicted variables in the between-person models [51]. As seen in Figure 3, the between-person model includes between-person means for both MVPA (µ_PA_) and pain(µ_P_), as well as random intercept variances. The two autoregressive terms (ϕ_1i_ and ϕ_4i_) and cross-lagged parameters (ϕ_3i_ and ϕ_2i_), also have fixed effects but their random effects are set to 0 [53]. Figure 4 includes the equations underlying both the within- and between-person models. As seen in Figure 5, marginalized status variables (race, ethnicity, gender, and SES) were entered into four separate models in addition to the main MVPA and pain model, to assess for moderation of the cross-lagged regression coefficients, specifically whether the presence of the moderator significantly changed the slope and path parameters.

#### Missing Data

Participants with at least 71% complete data (10 out of 14 days) were included in analyses (*n* = 251). Specifically, participants missing 5 or more days of either ActiGraph or pain survey data were excluded from analyses, resulting in an analytic sample size of 251. Chi-square tests were conducted to assess whether there were significant differences by dummy-coded sociodemographic groups in likelihood of meeting the 71% complete data threshold. Results indicated a significant relationship with SES and missing daily pain survey data, in which those with lower SES had more missing data than expected (χ^2^ (1, *N* = 261) = 6.24, *p* = 0.01). However, Cramer’s V effect size (0.16) demonstrates that this association is weak. Independent-samples *t*-tests were conducted to assess whether there were significant differences in primary outcomes (average daily pain and average daily MVPA of the complete study period) for participants who met the 71% complete data threshold. These results demonstrated that those who were who did not meet the threshold (*M* = 137.23, *SD* = 79.95) for complete ActiGraph data had significantly lower rates of average MVPA (*t*(261) = 3.00, *p* = 0.003) compared to those that met the threshold (*M* = 215.70, *SD* = 62.88). Cohen’s *d* (1.09) indicates that this is a large effect.

## 3. Results

### 3.1. Descriptive Data

#### 3.1.1. Pain

Among the analytic sample (*n* = 251), average total daily pain was 1.16 (*SD* = 1.03) out of a possible high score of 10. Average daily headaches were rated the highest (*M* = 0.32, *SD* = 0.34), followed by average daily back pain (*M* = 0.29, *SD* = 0.38), average daily arms, legs, joint pain (*M* = 0.25, *SD* = 0.34), stomach daily pain (*M* = 0.24, *SD* = 0.28), and chest daily pain (*M* = 0.06, *SD* = 0.12). These scores indicate that participants were within the range of “Not bothered at all” (0) to “Bothered a little” (1) by a given symptom per day. Independent-samples *t*-tests were run to assess for differences among sociodemographic groups on average reported daily pain. Participants who identified as white had significantly higher reports of average daily pain compared to participants of color (*t*(249) = 2.98, *p* = 0.003). The effect size (*d* = 0.38) indicates this is a small to medium effect. Additionally, those who identified as female had significantly higher daily pain ratings per day compared to male participants (*t*(244) = −2.07, *p* = 0.04). The effect size (*d* = 0.30) indicates this is also a small to medium effect. See Table 2 for pain averages across sociodemographic groups.

#### 3.1.2. Physical Activity

The average minutes spent in MVPA per day among the analytic sample was 215.96 (*SD* = 61.16). This average exceeds the physical activity national guidelines for adults [54], suggesting the current analytic sample is highly active. See Table 2 for MVPA averages across sociodemographic groups. Independent-samples *t*-tests were run to assess for differences among sociodemographic groups. No significant differences were found among groups on average time spent in MVPA per day.

### 3.2. Model Estimates

Five VAR(1) multilevel models were estimated in Mplus version 8 [55]. Models were run using Bayesian estimation and Markov chain Monte Carlo chains algorithms with seeds generated randomly. Non-informative priors were used as there are not previous DSEM analyses relating to the current study. Due to non-significant findings of cross-lagged regression parameters across all five models, models were conceptualized as stable according to potential scale reduction criterion (PSR). When PSR values were close to 1.001, indicating the total variance across the two of the MCMC was rendered similar to the variance of the within-chains [51], no additional iterations were added. For the main MVPA and pain model, 20,000 iterations were run with a thin of 100. For three of the moderation models testing the effect of race, ethnicity, and SES, 10,000 iterations were run with a thin of 100. For the gender moderation model, 30,000 iterations with a thin of 100 were needed to produce a PSR value of 1.001. For the SES moderation model, MVPA was defined by a constant (100) to reduce the variance between one and ten to attempt to remove a warning indicating difficulty computing the standardized estimates for clusters. This warning was present for all moderation models; however, defining by a constant did not remove the warning for SES.

The main model investigating the relationships among daily reported pain and MVPA (DIC = 48,990.26, pD = 709.11) did not reveal significant cross-lagged relationships. See Table 3 for full results. The subsequent moderation models testing the effects of race (moderation of previous-day MVPA on pain estimate = −0.03, *p* = 0.29; moderation of previous-day pain on MVPA estimate = −0.08, *p* = 0.38; DIC = 48,997.24, pD = 1001.26), ethnicity (moderation of previous-day MVPA on pain estimate = 0.01, *p* = 0.45; previous-day pain on MVPA = 0.14, *p* = 0.28; DIC = 48,999.97, pD = 1002.24), gender (moderation of previous-day MVPA on pain estimate = 0.03, *p* = 0.31; moderation of previous-day pain on MVPA estimate = −0.20, *p* = 0.21; DIC = 48,026.40, pD = 984.21), and SES (moderation of previous-day MVPA on pain estimate = 0.08, *p* = 0.32; moderation of previous-day pain on MVPA estimate = 0.001, *p* = 0.50; DIC = 17,707.90, pD = 808.91) on the cross-lagged relationships also did not render significant findings. All models demonstrated significant autoregressive relationships of the variables of interest (previous-day pain predicting next-day pain and previous-day MVPA predicting next-day MVPA); however, these findings are not surprising, as they demonstrate consistency among individuals and their symptoms and activity. Full moderation table results available upon request. Additional diagnostic information available upon request.

## 4. Discussion

The current study sought to understand the directionality of relationships among daily reported pain and actigraphy-measured physical activity across a 14 day longitudinal study period. DSEM analyses were implemented to examine the predictive ability of previous-day MVPA on next-day reported pain, in addition to previous-day reported pain on next-day MVPA. No significant cross-lagged relationships were found, suggesting that neither single directionality nor bidirectionality was present among these health indicators for this study sample of emerging adults in a university setting. These findings are inconsistent with research that has provided evidence for the deleterious effects of pain on physical activity [22,23], therapeutic effects of physical activity on pain [12,13], as well as the bidirectional effects of these constructs [43].

These relationships may not have been present in the study sample due to our measures of pain, our sample’s average reported pain, and non-chronic pain, community sample composition. Average reported pain per day in the sample was very low with low variability (*M* = 1.16, *SD* = 1.03). The current study used a composite score of total pain (stomach pain, back pain, pain in arms, legs, or joints, headaches, and chest pain) from the PHQ-15; however, when examining reported pain across pain symptoms, it is evident that headaches and back pain were endorsed the most for “bothering” participants. These pain symptoms are most commonly endorsed by this age group [5,7,8,9,56]. Future analyses may want to consider focusing on these pain symptoms exclusively and measuring multiple indicators, such as frequency, severity, and functional impairment on daily life, as they may be the most meaningful. A composite score including a count of pain locations like the one used in this study may not provide the best measure of sensitivity in pain symptoms for this population and may better serve clinical populations with specified pain locations. In fact, measures of internal consistency demonstrated poor validity among this sample, providing further evidence that most emerging adults in college likely are not experiencing multiple pain symptoms, but rather perhaps a few localized areas of pain, such as headache and back pain. More research focusing on these salient pain symptoms is needed to examine whether the relationships between pain and physical activity are present among non-clinical samples of emerging adults.

Descriptive analyses demonstrated this sample engages in a high amount of MVPA in reference to national guidelines for adults (*M* = 215.96, *SD* = 61.16), which recommend 150–300 min of moderate intensity or 75–150 min of vigorous intensity per week [54]. These findings suggest, despite varsity athletes being excluded from participation, our sample is unique with higher amounts of activity observed. Many studies have found the opposite [19,20,36], concluding that many emerging adults are not engaging in recommended activity, with one report suggesting this population is meeting less than half the recommended (2007) guidelines [18]. Some research among adolescents and emerging adults has demonstrated that wrist-worn accelerometers capture higher rates of activity compared to waist-worn accelerometers [42,57], which may have contributed to the high average.

Marginalized status was examined as a potential moderator in the relationships among pain and physical activity. Specifically, four moderation variables were entered into separate models testing the effects of gender, race, ethnicity, and SES separately on the cross-lagged relationships of physical activity and pain. The current study did not find any evidence for moderation of identity characteristics. Though no study to our knowledge has specifically looked at the moderating effects of identity among the relationships between pain and physical activity, there is evidence from other studies that marginalized groups are at an inequitable risk of worse pain experiences [8,26,27,28,29,31,32,34,58] and lower rates of physical activity [19,35,36,37,38].

Descriptively, we found significant mean differences in pain ratings by gender, with female identifying participants reporting higher average total pain than male participants—these findings are consistent with existing research [8,28,29]. In addition, there were significant differences in average total pain by race, with participants who identified as white with higher rates compared to individuals who identified as a person of color, though similar to gender, this effect was small to medium. This finding conflicts with some research finding the opposite phenomenon [26,27,58]. Perhaps this finding suggests that reports of pain are experienced differently than the impact of pain; other research has found that white adolescents also report more pain than those of color [34]. Findings from experimentally induced pain studies examining conditioned pain modulation, an identified risk factor of chronic pain, demonstrated that when engaging in similar levels of physical activity as their white peers, African American/Black and Latinx young adults exhibited the same conditioned pain responses [59,60]. These studies conjecture that if groups are similar in physical activity, they may share a similar risk to pain implying that physical activity may positively regulate pain processing among these groups [59,60]. Though this does not explain why we found higher averages of reported pain among white participants, perhaps physical activity is a protective factor against pain experiences (particularly because we had overall high averages of MVPA), weakening any moderation effect. More research needs to be done regarding the role of physical activity in moderating pain experiences.

Our study found no differences in rates of MVPA among sociodemographic groups, suggesting that individuals were engaging in similar levels of activity. This finding was surprising in light of research demonstrating disparate levels of activity across sociodemographic groups [19,35,36,37,38]. Some potential factors may have contributed to allowing for similar strength of the relationship between pain and physical activity among sociodemographic groups. For example, perhaps the centralization of physical activity resources and its access to all students (i.e., fitness center with various methods of engaging in MVPA, such as facilities with equipment and related programming) offset any differences among groups. Additionally, lack of moderation may have been due to campus location in a metropolitan area, in which participants walk across campus to various academic buildings and facilities, and commute by walking and biking, in addition to trains, busses, and cars. Lastly, although our sample was relatively diverse compared to other studies in this population, greater representation of participants of color—specifically having equal numbers of participation among different racial and ethnic groups, allowing differences to be assessed between each group—may provide even more power to examine a moderation effect.

### 4.1. Limitations and Future Directions

Though dummy coding sociodemographic variables lends itself to the current analyses due to small subgroup sample sizes, using dummy-coded identity variables is a reductionist way of viewing these relationships and potentially masks differences among subgroups (e.g., differences among Black/African American, American Indian/Alaskan Native, Asian or Asian American, Native Hawaiian/Pacific Islander, and those in the “other” category likely indicating multiracial status, as well as nuances among gender identifications (however, the current sample endorsed a small (*n* = 6) sample of non-binary individuals who were excluded from gender moderation analyses)). One aim of the current study attempted to explore whether these identity characteristics moderated daily health experiences. These identity characteristics should be studied in a more intersectional way, providing more accurate findings on the nuances of different identity characteristics of gender, race, ethnicity, and SES. In particular, it is essential to contextualize differences among groups with components of intersectionality [61]. Future studies should aim to include larger sample sizes with greater representation of participants in each demographic domain of these constructs to evaluate the relationships among pain and physical activity with an intersectional lens. Another approach may include focusing on specific populations and examining their unique experiences with pain and physical activity.

Another limitation includes missing data and associations with sample characteristics and outcome measures. Using a threshold of including participants who had at least 10 out of 14 days of complete data, analyses revealed that participants who were of lower SES had more data missing than expected (though this association was weak). In addition, those that were missing MVPA data had lower average MVPA observed. Taken together, perhaps individuals with lower SES were less likely to be included in analyses and decreased likelihood of finding moderation among pain and physical activity. Lastly, the current study only used daily cut offs of physical activity for inclusion in analyses (e.g., we included participants with at least 71% of days, or 10 out of 14 days). Some studies include an hourly threshold, with ranges of 7–10 h of activity per day to meet inclusion criteria [23,43,59,62,63,64], though they do not provide a rationale for these cut offs. To account for this, our MVPA calculations excluded non-wear time that was detected by the ActiGraph sensor and removed from computed physical activity data, as a selected feature of ActiLife (v6.13.4) software.

### 4.2. Strengths and Conclusions

A strength of the present study includes the use of objective accelerometer-measured physical activity over a two-week period, with daily diary reports of pain symptoms. These methods are an improvement over recall measures and suggest that future studies using other objective biometric measures are feasible as well. Regarding data, this study had excellent retention and little missing data, only 14 out of 265 participants were excluded from analyses due to not meeting the minimum daily data requirement. Given emerging adults’ comfort wearing wrist activity monitors and response to text messaging for daily diary completion, this study demonstrates a feasible method of examining these and related empirical questions with advanced statistical methods. Additionally, this is the first study of our knowledge using DSEM, a cutting-edge analytical approach, to explore the relationships among daily reported pain and actigraphy. DSEM has shown to be more sensitive to multiple data points per person. Future DSEM analyses investigating these concepts may want to consider using more than 14 points of data per person for each variable, such as using more moment-by-moment physical activity (e.g., minute-by-minute) and an objective indicator of pain experiences (e.g., heart rate or blood pressure). In addition, using an ecological momentary assessment of pain that collects more frequent assessments throughout the day may reduce likelihood of recall bias at the end of the day for daily data collection. Lastly, our sample was relatively diverse across several characteristics and allowed for tests of moderation of race, ethnicity, and SES when other studies may not have the power to do so.

In conclusion, the current study did not find evidence for significant predictive relationships among daily reported pain and observed physical activity. Despite these null findings, this study provided evidence for successful digital advancements in data collection of emerging adult samples with ActiGraph monitor wear and text reminders for daily survey completion. In addition, this relatively diverse study sample provided initial insight into the moderation of the relationships among pain and physical activity. Future research should aim to further include diverse samples of emerging adults and increased data points per person to investigate health concerns among this population.

## Figures and Tables

**Table 1:** Demographic Characteristics (*N* = 265).

		*n*	*%*
Gender			
	Male	76	28.68
	Female	183	69.06
	Other/Gender Non-Conforming/Queer	6	2.26
Race			
	White	141	53.21
	Black/African American	17	6.42
	American Indian/Alaskan Native	1	0.38
	Asian/Asian American	60	22.64
	Native Hawaiian/Pacific Islander	1	0.38
	Other	45	16.98
Ethnicity			
	Hispanic	62	23.40
	Non Hispanic	203	76.60
SES (Parent Income)			
	$0–$24,999	24	9.06
	$25,000–$49,999	41	15.47
	$50,000–$74,999	47	17.74
	$75,000–$99,999	48	18.11
	$100,000–$124,999	39	14.72
	$125,000–$149,999	20	7.55
	$150,000–$174,999	11	4.15
	$175,000–$199,999	10	3.77
	$200,000+	21	7.92

**Table 2:** Descriptive Results Pain and MVPA by Dummy-Coded Groups.

		Average Total Daily Pain	Average MVPA
		***M (SD)***	***M (SD)***
Race			
	White	1.33 (1.08) *	217.69 (64.86)
	Person of Color	0.95 (0.92)	213.96 (56.77)
Ethnicity			
	Hispanic	1.17 (0.97)	221.85 (52.02)
	Non Hispanic	1.15 (1.04)	214.16 (63.72)
Gender			
	Male	0.95 (0.90) *	213.41 (63.64)
	Female	1.25 (1.08)	217.53 (60.05)
SES (Parent Income)			
	$0–$49,999	1.19 (0.99)	209.32 (59.43)
	$50,000+	1.14 (1.04)	218.67 (62.02)

Significance * *p* < 0.05.

**Table 3:** MVPA Activity and Pain VAR (1) Model Standardized Results.

Level	Estimate	Standard Deviation	*p* Value (One-Tailed)	95% Credibility Interval
**Within**				
**Outcome: Pain**				
Predictor: Pain_t−1_	0.27	0.02	<0.001 *	0.23, 0.32
Predictor: MVPA_t−1_	0.02	0.02	0.15	−0.02, 0.06
**Outcome: MVPA**				
Predictor: MVPA_t−1_	0.14	0.02	<0.001 *	0.10, 0.18
Predictor: Pain_t−1_	−0.01	0.02	0.26	−0.05, 0.03
Covariance	−0.00	0.02	0.45	−0.04, 0.04
**Residual Variances**				
MVPA	0.98	0.01	<0.001 *	0.97, 0.99
Pain	0.92	0.01	<0.001 *	0.90, 0.95
**Between**				
Means				
MVPA	3.86	0.22	<0.001 *	3.45, 4.31
Pain	1.26	0.10	<0.001 *	1.08, 1.47
**Variances**				
MVPA	1.00	0.00	<0.001	1.00, 1.00
Pain	1.00	0.00	<0.001	1.00, 1.00

* Significant; Note. Bold heading demarcate sections for each outcome variable.

## Data Availability

The data presented in this study are available upon reasonable request from the corresponding author.
